# The Comparative Validity and Reproducibility of a Diet Quality Index for Adults: The Australian Recommended Food Score

**DOI:** 10.3390/nu7020785

**Published:** 2015-01-23

**Authors:** Clare E. Collins, Tracy L. Burrows, Megan E. Rollo, May M. Boggess, Jane F. Watson, Maya Guest, Kerith Duncanson, Kristine Pezdirc, Melinda J. Hutchesson

**Affiliations:** 1 Nutrition and Dietetics, School of Health Sciences, Faculty of Health and Medicine, University of Newcastle, Newcastle 2300, Australia; E-Mails: Tracy.Burrows@newcastle.edu.au (T.L.B.); Megan.rollo@newcastle.edu.au (M.E.R.); Jane.Watson@newcastle.edu.au (J.F.W.); Kerith.Duncanson@uon.edu.au (K.D.); Kristine.pezdirc@newcastle.edu.au (K.P.); Melinda.hutchesson@newcastle.edu.au (M.J.H.); 2 Priority Research Centre in Physical Activity and Nutrition, University of Newcastle, Newcastle 2300, Australia; E-Mail: Maya.Guest@newcastle.edu.au; 3 Occupational Health and Safety, School of Health Sciences, Faculty of Health and Medicine, University of Newcastle, Newcastle 2300, Australia; E-Mail: mboggess@asu.edu; 4 School of Mathematical and Statistical Sciences, College of Liberal Arts and Science, Arizona State University, Tempe, AZ 85287, USA

**Keywords:** diet quality index, validation, comparative validity, reproducibility, food frequency questionnaire, intra-class correlation coefficient, dietary methods

## Abstract

Adult diet quality indices are shown to predict nutritional adequacy of dietary intake as well as all-cause morbidity and mortality. This study describes the reproducibility and validity of a food-based diet quality index, the Australian Recommended Food Score (ARFS). ARFS was developed to reflect alignment with the Australian Dietary Guidelines and is modelled on the US Recommended Food Score. Dietary intakes of 96 adult participants (31 male, 65 female) age 30 to 75 years were assessed in two rounds, five months apart. Diet was assessed using a 120-question semi-quantitative food frequency questionnaire (FFQ). The ARFS diet quality index was derived using a subset of 70 items from the full FFQ. Reproducibility of the ARFS between round one and round two was confirmed by the overall intraclass correlation coefficient of 0.87 (95% CI 0.83, 0.90), which compared favourably to that for the FFQ at 0.85 (95% CI 0.80, 0.89). ARFS was correlated with FFQ nutrient intakes, particularly fiber, vitamin A, beta-carotene and vitamin C (0.53, 95% CI 0.37–0.67), and with mineral intakes, particularly calcium, magnesium and potassium (0.32, 95% CI 0.23–0.40). ARFS is a suitable brief tool to evaluate diet quality in adults and reliably estimates a range of nutrient intakes.

## 1. Introduction

The evaluation of relationships between health and intakes of single nutrients does not address the complexities of food and nutrient interactions in the human diet [1]. A focus solely on nutrients does not allow for assessment of the cumulative impact of nutrient interactions from a range of foods on health outcomes over time. Individuals do not usually consume single foods, but combinations of several foods and beverages that contain both nutritive and non-nutritive substances [2]. Given the complexity of assessing individual intakes, measurement of overall diet quality and variety by brief indices allows evaluation of several related aspects of dietary intake concurrently [3], and may provide a better measure of usual dietary intake patterns [4]. Diet quality refers to the nutritional adequacy of an individual’s dietary pattern and how closely this aligns with national dietary guidelines [3,5]. Scores or indexes of diet quality are being increasingly used in research as proxies for nutrient intakes, due to their lower researcher and respondent burden. The relationship between diet quality indices and nutritional adequacy, morbidity and mortality in adults has been reviewed [3,6]. This highlights that across these indices the risk for some health outcomes, including biomarkers of disease, incidence and risk of cardiovascular disease, some cancers and both cancer mortality and all-cause mortality can be quantified.

Diet quality indices have been derived by applying a scoring system to dietary intakes assessed by a variety of measures, including food frequency questionnaires (FFQ) and 24 h recalls. Indices are constructed by assigning higher scores within sub-scales based on more frequent or higher intakes of foods, nutrients, or both [3]. Generally there are two types of diet quality scores. These are either food-based or nutrient-based. A food-based diet quality index considers the number of foods or food groups consumed in a given period and assigns points based on diversity and/or frequency of intake [3,5], however no consideration is usually given to the sources or intakes of nutrients. Food-based scores rely on food consumption data only, meaning they can be scored quickly, but they typically have a limited food list and so may not fully reflect overall variety of foods consumed. This may be particularly for some population sub-groups, such as specific ethnic groups where food items may not have been included in the original FFQ food list. In comparison, nutrient-based scores require the dietary intake record to be analysed first in order to derive nutrient intakes, form which the diet quality scores can be calculated. For this reason food-based scores may be preferable for clinical settings and education purposes as they are more easily adapted to this purpose [3,6]. Given differences in food supply, consumption patterns and nutrition recommendations, diet quality indices should be country-specific.

The aim of this study was to evaluate the reproducibility of the Australian Recommended Food Score (ARFS) and its validity against a food frequency questionnaire from which it is derived.

## 2. Experimental Section

### 2.1. Subjects

Data from the Family Diet Quality Study was used in the current analysis. The methods have been published previously elsewhere [7]. Briefly, the population was healthy adults living full-time with at least one child aged 8 to 10 years, in New South Wales, Australia. Participants were recruited through a range of avenues, such as newspapers and school newsletters. Demographic and anthropometric data, together with the AES FFQ data scanned by an optical reader, were collected at baseline (September 2010–July 2011) and repeated at follow-up (January 2011–February 2012) 5 months later.

### 2.2. Australian Eating Survey Food Frequency Questionnaire (AES FFQ)

The AES FFQ was previously validated in adults [7] using standard adult portion sizes derived from unpublished data, purchased from the Australian Bureau of Statistics, from the National Nutrition Survey [8] and the “natural” serving size from standard items such as a slice of bread. The FFQ is a self-administered 120 item semi-quantitative FFQ that asks respondents to report usual dietary intake over the previous 6 months and takes approximately 30 min to complete [9,10,11,12,13,14,15,16]. It contains 15 supplementary questions (vitamin supplements, food behaviour, and sedentary behaviours). The FFQ has 24 questions on vegetables, 11 on fruit, nine on breads/cereals, nine on dairy foods, 32 on lunch/main meal food items, nine on beverages, 20 on snack foods/dessert and six on sandwich spreads/dressings and sauces. The response for each question is a frequency with options ranging from “never” to “≥7 times per day”. Nutrient intakes from the FFQ were computed from the AusNut 1999 database (All Foods) Revision 17 and AusFoods (Brands) Revision 5 [17] by summing over all food items, the product of the number of serves, the portion size in grams and the amount of the nutrient in a gram of that food.

### 2.3. Australian Recommended Food Score (ARFS)

The ARFS was modelled on the Recommended Food Score by Kant and Thompson [18] and the Australian Child and Adolescent Recommended Food Score (ACARFS) [19] as a brief food based diet quality index. The ARFS focuses on dietary variety within food groups recommended in the Australian Dietary Guidelines [20] for example the meat and alternatives food group encapsulates a range of differing foods each with unique nutrient profiles *i.e.*, red meat, fish, eggs, nuts and legumes. It takes approximately 10 min to complete and uses a sub-set of 70 AES FFQ questions. The ARFS has eight sub-scales with 20 questions related to vegetables, 12 to fruit, 7 to meat/flesh foods, six to non-meat/flesh protein foods, 12 to breads and cereals, 10 to dairy foods, one to water and two to spreads/sauces. Most foods are awarded one point for a consumption frequency of ≥once per week, which varies based on national dietary guidelines [20,21]. The ARFS score was calculated by summing the points for each item. The total score ranges from zero to 73 (Supplementary Table 1).

### 2.4. Statistical Methods

Food data were initially screened for implausible energy intakes however none were removed for this reason. Medians and interquartile ranges (IQR) were calculated for all nutrients. Univariate relationships were assessed using Fisher Exact tests to compare categorical variables by gender within data collection round, and exact symmetry tests [22] to compare categorical variables by gender on paired data. Continuous variables were similarly assessed using Wilcoxon rank-sum tests and Wilcoxon signed-rank tests for paired data.

*Reproducibility*: This was conducted separately for both the ARFS and the FFQ and evaluated by comparing the administration of the FFQ twice, five months apart using correlations and intra-class correlation coefficients (ICC) [23]. The ICC, the total variance, and its component parts, the within and the between person variance, are estimated using a linear regression model with a person-level random effect [24]. This model was bootstrapped [25] to obtain standard errors that accounted for the probable correlation between members of the same family.

*Validation:* The relationships between ARFS and FFQ nutrients and the percent energy (% E) from food groups were assessed using correlations, which were estimated by fitting a linear regression models to standardized variables, with standard errors clustered on family [26]. For the validation both rounds of measures were included which was possible due to aa random effects model being used. Total FFQ energy was included as an explanatory variable since both scores increase as the amount of foods increases.

Statistical significance is determined at the 5% level. Normality was visually checked where necessary using probability plots and box plots. Square root transforms were applied as necessary to improve the normality of the residuals of linear regression models. All nutrient calculations, data manipulation and statistical analysis was performed using Stata MP version 12.1 [27].

### 2.5. Ethics

This study was conducted according to the guidelines laid down in the Declaration of Helsinki and all procedures involving human subjects/patients were approved by the University of Newcastle Human Research Ethics Committee (Approval No. H-2010-1170). Written informed consent was obtained from all study participants.

## 3. Results

A total of 96 participants, from 68 separate families, completed FFQs at baseline and 68 at follow-up. Of these 67 completed the survey in both administration rounds. Thirty one participants were male and 65 were female in the initial administration round and of these 20 males and 48 females remained for round 2. Table 1 reports demographic and anthropometric variables for the two FFQ administration rounds and, by sex for *n* = 67 participants, Supplementary Table 2 provides details of all observations *n* = 151. There were no significant differences by sex or by administration round in education, smoking habits and general health. While there were some significant differences in weight, height, BMI and waist by sex, there were no significant differences in these variables between the two administrations rounds.

**Table 1:** Demographic and anthropometric data (151 observations on *N* = 67 participants (31 male) in 64 families). * Fisher’s exact test of homogeneity; † Wilcoxon rank-sum test for equality of populations; § No significant difference by gender in Round 1, Round 2 or in total according to the exact symmetry test of homogeneity for paired data; ** No significant difference by gender in Round 1, Round 2 or in total according to the Wilcoxon signed-rank test for equality of distributions on paired data.

	Round 1			Round 2		
	Male *N* = 20	Female *N* = 47	*p* *	Male *N* = 20	Female *N* = 47	*p* *
	*N* (%)	*N* (%)		*N* (%)	*N* (%)	
Education §						
Year 10	1 (5%)	3 (6%)		1 (5%)	3 (6%)	
Year 12	1 (5%)	7 (15%)		1 (5%)	7 (15%)	
Trade	3 (15%)	1 (2%)		5 (25%)	1 (2%)	
Certificate	4 (20%)	11 (23%)		2 (10%)	11 (23%)	
Degree	5 (25%)	13 (28%)		3 (15%)	14 (30%)	
Postgrad	6 (30%)	12 (26%)		8 (40%)	11 (23%)	
Total	20	47	0.44	20	47	0.03
Smoked within 10yrs §						
Yes	2 (10%)	2 (4%)		3 (15%)	3 (6%)	
No	18 (90%)	45 (96%)		17 (85%)	44 (94%)	
Total	20	47	0.58	20	47	0.35
Current Smoker §						
Yes	1 (5%)	0 (0%)		1 (5%)	0 (0%)	
No	19 (95%)	47 (100%)		19 (95%)	47 (100%)	
Total	20	47	0.30	20	47	0.30
General Health §						
Excellent	3 (33%)	6 (29%)		1 (14%)	8 (35%)	
Very Good	2 (22%)	11 (52%)		5 (71%)	11 (48%)	
Good	4 (44%)	4 (19%)		1 (14%)	4 (17%)	
Fair/Poor	0 (0%)	0 (0%)		0 (0%)	0 (0%)	
Total	9	21	0.25	7	23	0.62
	Median (IQR)	Median (IQR)	*p* †	Median (IQR)	Median (IQR)	*p* †
Age (years)	43.6 (41–47)	41.3 (38–45)	0.03	44.2 (41–47)	41.9 (39–46)	0.07
Height (cm) **	179 (174–182)	165 (162–170)	<0.01	179 (172–183)	164 (162–169)	<0.01
Weight (kg) **	81.7 (74–89)	64.9 (60–72)	<0.01	81.6 (74–91)	65.0 (60–73)	<0.01
BMI (kg/m^2^) **	25.7 (24–28)	23.5 (22–26)	0.06	26.8 (23–28)	23.5 (22–26)	0.12
Waist (cm) **	90.3 (84–98)	80.8 (74–86)	<0.01	91.4 (85–99)	80.4 (75–87)	<0.01

Table 2 reports the median FFQ nutrient intakes and the proportion of the sample by sex who met the Recommended Dietary Intake (RDI) targets. These results confirm that the sample is representative of the Australian adult population, having similar nutrient profiles as the last Australian National Nutrition Survey [28].

**Table 2:** Comparison of adult nutrient intakes, as assessed by the Australian Eating Survey (AES) food frequency questionnaire (FFQ), to Australian Recommended Dietary Intakes (RDI), Adequate Intake (AI) and upper limit, by gender.

Intake per day	Male (*N* = 31)			Female (*N* = 65)		
Meeting	RDI/AI	Median	Meeting RDI	RDI/AI	Median	Meeting RDI
Protein (g)	64	124.54	96%	46	92.25	95%
Fiber (g) AI	30	37.95	73%	25	28.41	70%
Vitamin A (µg)	900	1323.77	88%	700	1198.36	87%
Thiamine (mg)	1.2	2.27	90%	1.1	1.6	84%
Riboflavin (mg)	1.3	3.24	100%	1.1	2.42	97%
Niacin equiv. (mg)	16	56.95	100%	14	43.28	100%
Folate (µg)	420	468.17	65%	420	341.22	31%
Vitamin C (mg)	45	198.1	100%	45	174.38	98%
Calcium (mg)	1000	1375.59	71%	1000	1172.81	70%
Iron (mg)	8	19.09	100%	18	13.95	37%
Magnesium (mg)	420	540.95	75%	320	411.14	80%
Phosphorus(mg)	1000	2132.67	100%	1000	1642.88	95%
Potassium(mg) AI	3800	4447.83	73%	2800	3681.6	79%
Zinc (mg)	14	16.44	67%	8	13.14	82%
Exceeding	Upper Limit	Median	Exceeding Upper limit	Upper Limit	Median	Exceeding Upper limit
Sodium(mg)	920	2768.22	100%	920	2161.33	97%
% E Saturated fat	10	11	71%	10	13	79%

### 3.1. FFQ Reproducibility

Table 3 lists medians, correlations and intraclass correlation coefficients (ICC) for FFQ food group and nutrient intakes. Since observations for both administration rounds need to be present to estimate correlation between then, the number of observations available for use was only 67. When calculating the ICC however, all observations from both rounds can be utilized, thus the sample size was 163. The median correlation for nutrients was 0.72 (95% CI 0.51–0.92), which was attained by both thiamin and riboflavin. The least correlated was the percent energy (%E) from protein 0.49 (95% CI 0.19–0.78), and the most highly correlated was carbohydrate 0.83, (95% CI 0.68–0.98). We can expect tighter confidence intervals when using this approach. The median ICC was thiamin 0.73 (95% CI 0.55–0.80). The lowest ICC was the percent energy (% E) from protein 0.50 (95% CI 0.33–0.58), and the highest ICC was vitamin C, 0.88 (95% CI 0.92–0.93).

Data summarising the ARFS component subscales, the medians percentage energy from FFQ food groups are presented in Table 4. The median correlation was 0.66 (95% CI 0.48–0.84), which was attained by meat. The lowest correlation was for packaged snacks 0.52 (95% CI 0.32–0.72), and the most strongly correlated was for breakfast cereal 0.83, (95% CI 0.57–1.0). The median ICC was grains 0.62 (95% CI 0.53–0.70), with the lowest for condiments 0.44 (95% CI 0.28–0.61), and the highest for vegetables, 0.84% (95% CI 0.79–0.89).

**Table 3:** Reproducibility of Food Frequency Questionnaire (FFQ) nutrients: Median, interquartile range (IQR) and correlation, with 95% confidence interval, between round 1 and round 2.

	Round 1 *N* = 96		Round 2 *N* = 67		Correlation *N* = 67		ICC *N* = 163	
Nutrients/day	Median	IQR	Median	IQR	ρ	95% CI	ICC	95% CI
Energy								
Energy (kJ)	9601	(8024–11501)	8938	(7298–11085)	0.81	(0.67, 0.96)	0.85	(0.80, 0.89)
Protein (g)	101	(82–125)	96.5	(77.3–124.8)	0.65	(0.46, 0.84)	0.70	(0.62, 0.77)
Total fat (g)	75.5	(62.6–85.2)	73.6	(53.5–89.8)	0.71	(0.49, 0.93)	0.69	(0.61, 0.78)
Saturated fat (g)	30.1	(25.0–35.9)	30.6	(20.7–34.8)	0.67	(0.43, 0.90)	0.65	(0.55, 0.76)
Polyunsat. Fat (g)	9.7	(7.52–10.98)	9.15	(7.26–11.91)	0.76	(0.58, 0.94)	0.69	(0.63, 0.76)
Monounsat. Fat (g)	27.8	(22.9–31.7)	27.3	(19.6–35.5)	0.73	(0.52, 0.93)	0.72	(0.65, 0.79)
Cholesterol (mg)	283	(224–360)	252	(211–329)	0.66	(0.45, 0.87)	0.70	(0.60, 0.80)
Carbohydrate (g)	262	(217–341)	243	(192–337)	0.83	(0.68, 0.98)	0.85	(0.81, 0.89)
Sugars (g)	141	(100–182)	119	(97–168)	0.82	(0.68, 0.95)	0.83	(0.77, 0.90)
Alcohol (g)	12	(1.6–20.3)	8.14	(1.58–14.29)	0.79	(0.64, 0.95)	0.82	(0.75, 0.90)
Nutrients								
Fiber (g)	30.5	(23.8–37.4)	29.7	(23.9–35.6)	0.76	(0.65, 0.87)	0.79	(0.70, 0.87)
Vitamin A (µg)	1228	(1004–1511)	1225	(970–1667)	0.62	(0.36, 0.87)	0.69	(0.55, 0.83)
Retinol (µg)	297	(227–410)	317	(214–480)	0.69	(0.42, 0.97)	0.67	(0.57, 0.76)
Beta-carotene(µg)	5316	(3997–6581)	5122	(3824–6959)	0.61	(0.35, 0.88)	0.72	(0.54, 0.89)
Thiamin (mg)	1.77	(1.41–2.21)	1.74	(1.38–2.16)	0.72	(0.52, 0.92)	0.73	(0.66, 0.80)
Riboflavin (mg)	2.59	(2.10–3.19)	2.54	(2.06–3.34)	0.72	(0.51, 0.92)	0.72	(0.66, 0.78)
Niacin (mg)	45.3	(38.8–55.7)	43.8	(36.4–54.7)	0.70	(0.51, 0.88)	0.74	(0.67, 0.81)
Vitamin C (mg)	184	(140–235)	167	(133–213)	0.81	(0.61, 0.101)	0.88	(0.82, 0.93)
Folate (µg)	372	(288–455)	357	(279–459)	0.78	(0.62, 0.93)	0.80	(0.74, 0.85)
Calcium (mg)	1200	(949–1413)	1205	(903–1603)	0.72	(0.55, 0.89)	0.71	(0.63, 0.79)
Iron (mg)	15.1	(11.5–18.1)	14.3	(11.2–17.7)	0.75	(0.60, 0.91)	0.76	(0.70, 0.83)
Magnesium (mg)	450	(371–531)	430	(344–541)	0.83	(0.70, 0.95)	0.85	(0.80, 0.90)
Phosphorus(mg)	1743	(1421–2148)	1704	(1273–2256)	0.73	(0.57, 0.89)	0.75	(0.68, 0.82)
Potassium(mg)	3881	(3247–4610)	3730	(3093–4580)	0.73	(0.58, 0.88)	0.78	(0.72, 0.83)
Sodium(mg)	2272	(1783–2846)	2313	(1765–2865)	0.76	(0.58, 0.93)	0.80	(0.75, 0.85)
Zinc (mg)	13.9	(11.3–17.2)	13.5	(11.1–16.4)	0.71	(0.54, 0.87)	0.75	(0.68, 0.81)
Water (mL)	3469	(2977–4024)	3388	(2987–3837)	0.80	(0.64, 0.96)	0.87	(0.83, 0.91)
Percent Energy								
Protein	18	(16.0–20.0)	18	(16.0–20.0)	0.49	(0.19, 0.78)	0.50	(0.33, 0.68)
Carbohydrate	47.5	(44.0–52.5)	48	(43.0–52.0)	0.68	(0.50, 0.87)	0.66	(0.58, 0.74)
Total Fats	30	(27.0–33.0)	30	(28.0–34.0)	0.64	(0.47, 0.81)	0.60	(0.50, 0.69)
Saturated Fat	12	(11.0–14.0)	12	(11.0–14.0)	0.64	(0.42, 0.85)	0.63	(0.55, 0.72)
Alcohol	4	(0.50–6.00)	2	(1.00–5.00)	0.77	(0.63, 0.91)	0.78	(0.71, 0.85)
Percent Fat								
Saturated	45	(42.0–49.0)	45	(42.0–48.0)	0.72	(0.56, 0.89)	0.73	(0.64, 0.81)
Polyunsaturated	14	(12.0–15.5)	15	(13.0–16.0)	0.80	(0.59, 0.102)	0.76	(0.68, 0.84)
Monounsaturated	41	(39.0–43.0)	41	(39.0–42.0)	0.57	(0.36, 0.79)	0.64	(0.51, 0.76)

### 3.2. ARFS Reproducibility

The median correlation between the two rounds for ARFS food groups was 0.66 (95% CI 0.48–0. 84), which was attained by meat (Table 4). The lowest correlation was for was vegetables, 0.59 (95% CI 0.34–0.83), and the strongest for ARFS total score, 0.83 (95% CI 0.68–0.98). Similarly, the median ICC was thiamin 0.69 (95% CI 0.55–0.80). The lowest ICC was for meat, 0.62 (95% CI 0.51–0.73), and the highest ICC was for ARFS total score, 0.87 (95% CI 0.83–0.90).

**Table 4:** Reproducibility of The Australian Recommended Food Score (ARFS) components and the AES FFQ percentage of energy (%E) from core and non-core food groups: Median, interquartile range (IQR) and correlation between rounds.

Scores	Round 1 *N* = 96		Round 2 *N* = 67		Correlation *N* = 67		ICC *N* = 163	
ARFS (max avail. score)	Median	IQR	Median	IQR	*ρ*	95% CI	ICC	95% CI
ARFS total(73)	36	(32.0–42.5)	35	(31.0–41.0)	0.83	(0.68, 0.98)	0.87	(0.83, 0.90)
Vegetables(21)	14	(12.0–16.0)	13	(11.0–15.0)	0.59	(0.34, 0.83)	0.69	(0.58, 0.80)
Fruit(12)	7	(4.0–8.0)	6	(4.0–8.0)	0.64	(0.47, 0.81)	0.68	(0.61, 0.75)
Meat(7)	2	(2.0–3.0)	2	(1.0–3.0)	0.66	(0.48, 0.84)	0.62	(0.51, 0.73)
Meat alternatives(6)	2	(1.0–3.0)	2	(1.0–3.0)	0.78	(0.62,0. 93)	0.79	(0.72, 0.86)
Grains(13)	6	(4.0–7.0)	6	(5.0–7.0)	0.64	(0.48, 0.80)	0.68	(0.59, 0.77)
Dairy(11)	5	(3.0–6.0)	5	(4.0–6.0)	0.77	(0.63, 0.91)	0.79	(0.73, 0.84)
Extras(2)	1	(0.0–1.0)	1	(0.0–1.0)	0.65	(0.44, 0.85)	0.66	(0.56, 0.76)
%E from food groups								
FFQ CORE	67.5	(58.0–76.0)	69	(60.0–75.0)	0.71	(0.51, 0.91)	0.76	(0.68, 0.85)
Vegetables	8	(6.0–11.0)	8	(6.0–10.0)	0.79	(0.66, 0.93)	0.84	(0.79, 0.89)
Fruit	8	(5.0–11.5)	8	(5.0–11.0)	0.60	(0.46, 0.74)	0.57	(0.39, 0.74)
Meat	11.5	(8.0–15.0)	11	(7.0–14.0)	0.53	(0.15, 0.91)	0.52	(0.31, 0.74)
Meat alternatives	4	(2.0–7.0)	5	(2.0–7.0)	0.53	(0.26, 0.80)	0.57	(0.42, 0.71)
Grains	22	(15.0–27.0)	22	(18.0–25.0)	0.60	(0.45, 0.76)	0.62	(0.53, 0.70)
Dairy	9	(7.0–14.0)	11	(7.0–16.0)	0.54	(0.32, 0.76)	0.52	(0.39, 0.64)
FFQ NON-CORE	32.5	(24.0–42.0)	31	(25.0–40.0)	0.71	(0.51, 0.91)	0.77	(0.69, 0.84)
Sweet drinks, fruit juice	1	(0.0–4.0)	1	(0.0–4.0)	0.78	(0.59, 0.97)	0.78	(0.70, 0.87)
Packaged snacks	1	(0.5–3.5)	1	(0.0–3.0)	0.52	(0.32, 0.72)	0.56	(0.38, 0.74)
Confectionary	4	(2.0–7.0)	3	(1.0–6.0)	0.63	(0.47, 0.78)	0.54	(0.41, 0.67)
Baked sweet products	4	(2.0–7.0)	3	(2.0–7.0)	0.76	(0.62, 0.90)	0.72	(0.58, 0.85)
Take-away	6	(4.0–8.0)	6	(4.0–8.0)	0.77	(0.53, 0.100)	0.76	(0.69, 0.84)
Condiments	2	(1.0–3.5)	2	(1.0–5.0)	0.60	(0.38, 0.82)	0.44	(0.28, 0.61)
Processed fatty meats	2	(1.0–3.0)	2	(1.0–3.0)	0.69	(0.50, 0.88)	0.57	(0.39, 0.76)
Breakfast cereal	7	(4.0–10.0)	8	(5.0–11.0)	0.83	(0.57, 0.100)	0.70	(0.58, 0.82)
Meat meals with veg.	7	(4.5–10.0)	6	(4.0–9.0)	0.52	(0.19, 0.85)	0.54	(0.36, 0.71)
Meat meals no veg.	1	(0.0–2.0)	1	(0.0–1.0)	0.53	(0.26, 0.79)	0.54	(0.43, 0.65)

### 3.3. Validity between ARFS and FFQ

Table 5 summarises the correlations between the ARFS sub-scale components and FFQ nutrients adjusted for total FFQ energy, significant at the 5% level. Negative correlations were found for % energy from saturated fat and ARFS total score and ARFS components of fruit, vegetables and grains, this is likely as foods high in SFA are not accounted for in ARFS so as the total ARFS increases intake of SFA decreases. ARFS was highly correlated with FFQ nutrient intakes, particularly for fiber, 0.38 (95% CI 0.27–0.49); vitamin A, 0.45 (95% CI 0.23–0.61); beta-carotene, 0.51 (95% CI 0.34–0.69); and vitamin C, 0.53 (95% CI 0.37–0.67). There were also strong correlations with mineral intakes, particularly calcium, 0.23 (95% CI 0.10–0.46); magnesium, 0.30 (95% CI 0.21–0.40); and potassium, 0.32 (95% CI 0.23–0.40) (See Supplementary Figure 1).

**Table 5:** Correlations between the Australian Recommended Food Score (ARFS) and the Australian Eating Survey (AES) FFQ components, adjusted for total FFQ energy, significant at the 5% level. Shaded cells are negative correlations.

	ARFS Total	ARFS Veg	ARFS Fruit	ARFS Meat	ARFS Meat Alt	ARFS Grains	ARFS Dairy	ARFS Extra
Protein (g)				0.19		0.10	0.22	
Saturated fat (g)		−0.09	−0.13				0.14	
Cholesterol (mg)				0.26			0.21	
Carbohydrate (g)				−0.09	−0.09		−0.07	
Sugars (g)			0.15		−0.14			
Fiber (g)	0.38	0.31	0.37		0.25	0.16		
Vitamin A (µg)	0.45	0.38	0.37		0.29			
Retinol (µg)								
Beta-carotene(µg)	0.51	0.43	0.47		0.30			
Thiamine (mg)								0.17
Riboflavin (mg)	0.16					0.14	0.24	
Niacin equiv. (mg)	0.12			0.20		0.09	0.12	
Folate (µg)	0.27	0.20	0.19		0.15	0.17		
Vitamin C (mg)	0.53	0.49	0.51	0.16	0.22			
Calcium (mg)	0.23					0.15	0.40	
Iron (mg)	0.12					0.13		
Magnesium (mg)	0.30	0.20	0.22		0.19	0.18	0.15	−0.10
Phosphorus (mg)	0.32	0.24	0.28	0.15	0.13	0.12	0.20	−0.13
Potassium(mg)	0.32			0.09		0.14	0.27	−0.07
Sodium(mg)			−0.12					0.13
Zinc (mg)				0.13			0.17	
% E Saturated Fat	−0.23	−0.22	−0.29			−0.20		0.18

Table 6 displays the correlations between the ARFS components and FFQ nutrients, adjusted for total FFQ energy, significant at the 5% level. There were significant, strong correlations between the corresponding ARFS and FFQ food groups vegetables, fruit, meat, meat alternatives, grains and dairy (0.50, 0.68, 0.42, 0.56, 0.28, 0.46, respectively) (See Supplementary Figure 2).

ARFS was strongly positively correlated with FFQ %E food group intakes, particularly for fruit, 0.38 (95% CI 0.27–0.49); vegetable, 0.45 (95% CI 0.23–0.61), meat alternatives, 0.51 (95% CI 0.34–0.69); and dairy, 0.53 (95% CI 0.37–0.67). There were also strong correlations with mineral intakes, particularly calcium, 0.23 (95% CI 0.10–0.46); magnesium, 0.30 (95% CI 0.21–0.40); and potassium, 0.32 (95% CI 0.23–0.40).

**Table 6:** Correlations between the Australian Recommended Food Score (ARFS) and the Australian Eating Survey (AES) FFQ food groups, adjusted for total FFQ energy, significant at the 5% level. Light grey shaded cells are those with the same group in row and column where positive correlation would be anticipated. Dark grey shaded cells are negative correlations.

Percentage of Energy From	ARFS Total	ARFS Veg	ARFS Fruit	ARFS Meat	ARFS Meat Alt	ARFS Grains	ARFS Dairy	ARFS Extra
CORE	0.31	0.30	0.32		0.25			−0.26
Vegetables	0.22	0.50	0.20					
Fruit	0.37	0.33	0.68					
Meat				0.42	−0.30			
Meat alternatives	0.31	0.28	0.23		0.56			
Grains						0.28		
Dairy	0.23					0.21	0.46	
NON-CORE	−0.31	−0.30	−0.32		−0.25			0.26
Sweet drinks, fruit juice	−0.25	−0.27	−0.18		−0.19			
Packaged snacks		−0.20			−0.17			
Confectionary		−0.25			−0.23			
Baked sweet products			−0.24	−0.26				0.19
Take-away	−0.29	−0.26	−0.25			−0.26		
Condiments					0.21			0.40
Processed fatty meats			−0.26		−0.27			
Breakfast cereal						0.23		−0.21
Meat meals with vegetables				0.32	−0.26			
Meat meals without vegetables								0.15

## 4. Discussion

The reproducibility and comparative validity of the ARFS in Australian adults was assessed in the current study by comparing food and nutrient intake data from the AES FFQ over two administration rounds five months apart, to estimate intra-class correlation coefficients (ICC). The reproducibility of the ARFS was confirmed as shown by ICCs for each nutrient assessed as being similar to those for the AES FFQ. The median ICC for ARFS nutrients was 0.66 (0.48–0.84) was similar when compared with the median ICC for FFQ nutrients 0.72 (0.51–0.92). These results confirm that the ARFS can be used when a brief evaluation of overall diet quality is required and with the advantages of considerably lower participant and researcher burden compared to other methods of dietary intake assessment.

ARFS was found to be highly correlated with FFQ nutrient intakes, particularly fiber, vitamin A, beta-carotene, and vitamin C. There were also strong positive correlations with mineral intakes for calcium, magnesium and potassium. These results indicate that the ARFS reflects the intake of a variety of nutrients which are known to be associated with health outcomes. These results are similar to a larger validation study in 6542 adults by Toft *et al.* [29], that used an FFQ to validate a food based diet quality score for fiber and vitamin C. However correlations in our study were higher for calcium (0.23), magnesium (0.30) and vitamin A (0.45) [29]. In the present study there were significant and positive correlations between the corresponding ARFS sub-scale score and the corresponding FFQ food groups of vegetables, fruit, meat and vegetarian alternatives, grains and dairy. This was not completely expected as although the ARFS score is based on sub-set of FFQ questions, only nutrient dense foods and drink are included. The approach to scoring is also different with the ARFS being a simple count based on foods usually consumed at least weekly, while the FFQ incorporates the total number of daily serves, portion size and nutrient content. Similar correlations have been previously found between fruits and vegetables assessed by FFQ and diet quality scores [29]. Toft *et al*. [29] found correlations with grams of fruit (*r* 0.55) and vegetables (*r* 0.48) and in the current study, *r* = 0.68 and 0.50 respectively. These results suggest that the ARFS does reflect intakes across a variety of nutrients. In addition to food groups and that the foods included in the ARFS are representative of the AES.

The correlation coefficients from the current study are comparable to those found in the validation study conducted in children and adolescents [9]. This was anticipated given that adult AES FFQ was modified from the child and adolescent version [9] and both studies had a similar design. When the results of the current analysis are compared to those in children and adolescents, which reported a median energy adjusted correlation between FFQ and food records of 0.32, the median correlation of all nutrients in the current study are stronger at 0.72, suggesting that frequency based on weekly consumption of a range of nutrient-dense foods is a stronger predictor of nutrient intakes in adults compared to children.

For dietary instruments to be used to examine associations between diet and disease outcomes, it is suggested that correlations between the instrument and the reference method need to be in the range of at least 0.3 or 0.4 [30]. The current study found correlations significantly greater than 0.3 for all nutrients indicating that the ARFS is an appropriate tool to assess dietary patterns and that it has the potential to be used to evaluate relationships between diet and health status.

The ARFS food based diet quality score accounts for diet variety, particularly fruits and vegetables and assesses the healthiness of diet in relation to National Dietary guidelines however does not account for non-core foods. The ARFS has application as a brief tool to assess overall diet quality and provide a cross-sectional snapshot of dietary intake in relation to dietary guidelines and dietary compliance however may not be sensitive to detect change over time.

A general limitation of validation studies is that the results are not necessarily transferable to other populations. This is generally due to the dietary assessment method such as an FFQ being based on the local food supply and portion size data from national-level surveys [31]. A sample size of at least 50 is desirable for each demographic group [32], and ideally between 100 and 200 participants [33]. Although the sample size in the present study was adequate at the group level it was inadequate to confirm validity and reproducibility for subsets based on age, ethnicity or BMI category. The current sample included 65 female (68%) and therefore results likely to represent females as sub-category, but not males, however all participants are parents of primary school aged children so more likely to reflect a younger age group of adults. Performance of the AES FFQ also needs to be evaluated in populations of varying socioeconomic status and ethnicity. Strengths of the current study include that data were screened for implausible intakes. The reporting period of the FFQ was the previous six months so is likely to reflect differing intake due to seasonality. Lastly, by using statistical methods appropriate for repeated measures and correlated data, that is bootstrapped ICC, strong correlations were revealed.

## 5. Conclusions

This study demonstrated that the Australian Recommended Food Score diet quality index is acceptable in classifying participants into quintiles of nutrient and food intakes. The ARFS was found to be reproducible over a five month period and provides an important contribution to the diet quality indices available for assessing usual intakes in adults. Further research is required to evaluate it use in clinical practice, epidemiologic research and public health interventions in terms of evaluating dietary change and predicting disease risk and evaluating in more diverse populations such as older Australians.

## Supplementary Files

Supplementary File 1
