# Supplementary material for: The Comparative Validity and Reproducibility of a Diet Quality Index for Adults: The Australian Recommended Food Score
Source: Nutrients. 2015 Jan 23;7(2):785–98. doi: 10.3390/nu7020785 (PMC4344560; doi:10.3390/nu7020785)
Supplement: Supplementary File 1 [file nutrients-07-00785-s001.docx]

**Supplementary Material**

**Figure S1.** Correlations between the Australian Recommended Food Score (ARFS) and the Australian Eating Survey (AES) FFQ, adjusted for total energy, with 95% confidence intervals.

**Figure S2.** Correlations between the Australian Recommended Food Score (ARFS) and the Australian Eating Survey (AES) FFQ food groups, adjusted for total FFQ energy, with 95% confidence intervals.

**Table S1.** The Australian Recommended Food Score (ARFS) questions and scoring.

| **Category** | **Question** | **Frequency** | **Points** |
| --- | --- | --- | --- |
| Vegetables | 1 How many times a week do you eat vegetables with your meal at night? (not including hot chips) | 3–4 times/week | 1 |
|  |  | or ≥5 times/week | 2 |
|  | 2 Potato (boiled, mashed, baked) | ≥1 times per week | 1 |
|  | 3 Pumpkin | ≥1 times per week | 1 |
|  | 4 Sweet potato | ≥1 times per week | 1 |
|  | 5 Cauliflower | ≥1 times per week | 1 |
|  | 6 Green beans | ≥1 times per week | 1 |
|  | 7 Spinach | ≥1 times per week | 1 |
|  | 8 Cabbage or brussels sprouts | ≥1 times per week | 1 |
|  | 9 Peas | ≥1 times per week | 1 |
|  | 10 Broccoli | ≥1 times per week | 1 |
|  | 11 Carrots | ≥1 times per week | 1 |
|  | 12 Zucchini, eggplant, squash | ≥1 times per week | 1 |
|  | 13 Capsicum | ≥1 times per week | 1 |
|  | 14 Corn, sweet corn, corn on the cob | ≥1 times per week | 1 |
|  | 15 Mushrooms | ≥1 times per week | 1 |
|  | 16 Tomatoes | ≥1 times per week | 1 |
|  | 17 Lettuce | ≥1 times per week | 1 |
|  | 18 Celery, cucumber | ≥1 times per week | 1 |
|  | 19 Avocado | ≥1 times per week | 1 |
|  | 20 Onion, spring onion, leek | ≥1 times per week | 1 |
|  | Maximum possible Vegetable score |  | 21 |
| Fruit | 21 How many pieces of fruit do you eat? | ≥1 times per day | 1 |
|  | 22 Canned fruit e.g., peaches, Two fruits | ≥1 times per week | 1 |
|  | 23 Fresh fruit salad | ≥1 times per week | 1 |
|  | 24 Dried fruit e.g., sultanas, dried apricots | ≥1 times per week | 1 |
|  | 25 Apple or pear | ≥1 times per week | 1 |
|  | 26 Orange, mandarin, grapefruit | ≥1 times per week | 1 |
|  | 27 Banana | ≥1 times per week | 1 |
|  | 28 Peach, nectarine, plum or apricot | ≥1 times per week | 1 |
|  | 29 Mango or paw-paw | ≥1 times per week | 1 |
|  | 30 Pineapple | ≥1 times per week | 1 |
|  | 31 Grapes, strawberries, blueberries | ≥1 times per week | 1 |
|  | 32 Melon e.g., watermelon, rockmelon | ≥1 times per week | 1 |
|  | Maximum possible Fruit score |  | 12 |
| Category | Question | Frequency | Points |
| Meat | 33 Mince e.g., spaghetti bolognese, rissoles, lasagne | ≥never & ≤ 1/month | 1 |
|  | 34 Meat (beef or lamb) roast, chops(with/without sauce) | 1–4 serves per week | 1 |
|  | 35 Chicken e.g., BBQ, satay, stir fry (with/without sauce) | 1–4 serves per week | 1 |
|  | 36 Pork e.g., chops, sweet & sour (with/without sauce) | 1–4 serves per week | 1 |
|  | 37 Fresh fish, not crumbed or battered | ≥1 times per week | 1 |

**Table S1.** *Cont.*

| **Category** | **Question** | **Frequency** | **Points** |
| --- | --- | --- | --- |
|  | 38 Canned tuna, salmon, sardines | ≥1 times per week | 1 |
|  | 39 Other seafood e.g., prawns, lobster | ≥1 times per week | 1 |
|  | Maximum possible Meat score |  | 7 |
| Meat | 40 Nuts e.g., peanuts, almonds | ≥1 times per week | 1 |
| Alternative | 41 Peanut butter, Nutella | ≥1 times per week | 1 |
|  | 42 Eggs e.g., boiled, scrambled | ≥1 times per week | 1 |
|  | 43 Soybeans, tofu | ≥1 times per week | 1 |
|  | 44 Baked beans | ≥1 times per week | 1 |
|  | 45 Other beans, lentils e.g., chickpeas, split peas | ≥1 times per week | 1 |
|  | Maximum possible Meat Alternatives score |  | 6 |
| Grain | 46 What type of bread do you usually eat? | Brown, multigrain | 2 |
|  |  | Other e.g., Rye, or | 1 |
|  | 47 Muesli | ≥1 times per week | 1 |
|  | 48 Porridge | ≥1 times per week | 1 |
|  | 49 Breakfast cereal e.g., Weet-bix, Nutri-grain | ≥1 times per week | 1 |
|  | 50 Bread, pita bread, roll or toast | ≥1 times per week | 1 |
|  | 51 English muffin, bagel or crumpet | ≥1 times per week | 1 |
|  | 52 Rice | ≥1 times per week | 1 |
|  | 53 Other grains e.g., cous cous, burghul | ≥1 times per week | 1 |
|  | 54 Noodles e.g., egg noodles | ≥1 times per week | 1 |
|  | 55 Pasta e.g., spaghetti, lasagne, pasta bake | ≥1 times per week | 1 |
|  | 56 Clear soup with rice or noodles | ≥1 times per week | 1 |
|  | 57 Tacos, burritos, enchiladas | ≥1 times per week | 1 |
|  | Maximum possible Grain score |  | 13 |
| Dairy | 58 How often: glass of milk, tub of yoghurt, slice cheese? | ≥2 times per day | 1 |
|  | 59 What type of milk do you usually drink? | Regular milk, or | 1 |
|  |  | Low fat or soy milk | 2 |
|  | 60 Flavoured milk e.g., Moove, Oak, hot chocolate | ≥1 serves per week | 1 |
|  | 61 Plain milk-glass or with cereal | ≥1 serves per week | 1 |
|  | 62 Ice cream-vanilla, chocolate, strawberry | ≥1 serves per week | 1 |
|  | 63 Frozen yoghurt | ≥1 serves per week | 1 |
|  | 64 Yoghurt (not frozen) plain or flavoured | ≥1 serves per week | 1 |
|  | 65 Cottage cheese or ricotta | ≥1 serves per week | 1 |
|  | 66 Cheese, including on sandwiches, biscuits, toast | ≥1 serves per week | 1 |
|  | 67 Cheese spread, cream cheese | ≥1 serves per week | 1 |
|  | Maximum possible Dairy Food score |  | 11 |
| Water | 68 Water-bottled, tap, unflavoured mineral water | ≥4 times per day | 1 |
|  | Maximum possible Water score |  | 1 |
| Extra | 69 Vegemite, Mighty Mite, Promite, Marmite | ≥1 times per week | 1 |
|  | 70 Tomato sauce, barbecue sauce | ≥1 times per week | 1 |
|  | Maximum possible Extras score |  | 2 |
|  | Maximum possible TOTAL SCORE |  | 73 |

**Table S2.** Demographic and anthropometric data (151 observations on *N* = 95 participants
(31 male) in 64 families). ***** Fisher’s exact test of homogeneity; † Wilcoxon rank-sum test for equality of populations; § No significant difference by gender in Round 1, Round 2 or in total according to the exact symmetry test of homogeneity for paired data; ** No significant difference by gender in Round 1, Round 2 or in total according to the Wilcoxon signed-rank test for equality of distributions on paired data.

|  | **Round 1** | | | | **Round 2** | | |
| --- | --- | --- | --- | --- | --- | --- | --- |
|  | **Male *N* = 31** | | **Female *N* = 65** |  | **Male *N* = 20** | **Female *N* = 48** |  |
|  | ***N* (%)** | | ***N* (%)** | ***p* *** | ***N* (%)** | ***N* (%)** | ***p* *** |
| Education § | | | | | | | |
| Year 10 | 2 (6%) | | 5 (8%) |  | 1 (5%) | 3 (6%) |  |
| Year 12 | 1 (3%) | | 9 (14%) |  | 1 (5%) | 7 (15%) |  |
| Trade | 5 (16%) | | 2 (3%) |  | 5 (25%) | 1 (2%) |  |
| Certificate | 7 (23%) | | 16 (25%) |  | 2 (10%) | 11 (23%) |  |
| Degree | 8 (26%) | | 20 (31%) |  | 3 (15%) | 14 (30%) |  |
| Postgrad | 8 (26%) | | 12 (19%) |  | 8 (40%) | 11 (23%) |  |
| Total | 31 | | 64 | 0.18 | 20 | 47 | 0.03 |
| Smoked within 10yrs § | | | | | | | |
| Yes | 2 (6%) | | 4 (6%) |  | 3 (15%) | 3 (6%) |  |
| No | 29 (94%) | | 61 (94%) |  | 17 (85%) | 45 (94%) |  |
| Total | 31 | | 65 | 1 | 20 | 48 | 0.35 |
| Current Smoker § | | | | | | | |
| Yes | 1 (3%) | | 2 (3%) |  | 19 (95%) | 48 (100%) |  |
| No | 30 (97%) | | 62 (97%) |  | 1 (5%) | 0 (0%) |  |
| Total | 31 | | 64 | 1 | 20 | 48 | 0.3 |
| General Health § | | | | | | | |
| Excellent | 4 (33%) | 6 (21%) | |  | 1 (14%) | 7 (35%) |  |
| Very Good | 3 (25%) | 15 (54%) | |  | 5 (71%) | 11 (48%) |  |
| Good | 5 (42%) | 7 (25%) | |  | 1 (14%) | 4 (17%) |  |
| Fair/Poor | 0 (0%) | 0 (0%) | |  | 0 (0%) | 0 (0%) |  |
| Total | 12 | 28 | | 0.25 | 7 | 23 | 0.62 |
|  | Median (IQR) | Median (IQR) | | *p* † | Median (IQR) | Median (IQR) | *p* † |
| Age (years) | 44.9 (41–48) | 41.3 (39–45) | | 0.01 | 44.2 (41–47) | 41.9 (39–46) | 0.07 |
| Height (cm) ** | 179 (174–183) | 165 (162–169) | | 0 | 179 (172–183) | 164 (162–169) | 0 |
| Weight (kg) ** | 82.6 (76–91) | 65.6 (60–72) | | 0 | 81.6 (74–91) | 65.0 (60–73) | 0 |
| BMI (kg/m^2^) ** | 26.2 (24–29) | 24.0 (2–27) | | 0.01 | 26.8 (23–28) | 23.5 (22–26) | 0.12 |
| Waist (cm) ** | 92.0 (84–100) | 81.0 (75–86) | | 0 | 91.4 ( 85–99) | 80.4 (75–87) | 0 |

© 2015 by the authors; licensee MDPI, Basel, Switzerland. This article is an open access article distributed under the terms and conditions of the Creative Commons Attribution license (http://creativecommons.org/licenses/by/4.0/).
